# Optical Fiber Nanotips Coated with Molecular Beacons for DNA Detection

**DOI:** 10.3390/s150509666

**Published:** 2015-04-24

**Authors:** Ambra Giannetti, Andrea Barucci, Franco Cosi, Stefano Pelli, Sara Tombelli, Cosimo Trono, Francesco Baldini

**Affiliations:** 1 CNR—Institute of Applied Physics “Nello Carrara”, Via Madonna del Piano 10, 50019 Sesto Fiorentino (FI), Italy; E-Mails: a.barucci@ifac.cnr.it (A.B.); f.cosi@ifac.cnr.it (F.C.); s.pelli@ifac.cnr.it (S.P.); s.tombelli@ifac.cnr.it (S.T.); c.trono@ifac.cnr.it (C.T.); f.baldini@ifac.cnr.it (F.B.); 2 Museo Storico della Fisica e Centro Studi e Ricerche Enrico Fermi, Piazza del Viminale 1, 00184 Rome, Italy

**Keywords:** molecular beacon, optical fiber nanotip, nanosensor, optical biosensors, fluorescence, mRNA, DNA, survivin

## Abstract

Optical fiber sensors, thanks to their compactness, fast response and real-time measurements, have a large impact in the fields of life science research, drug discovery and medical diagnostics. In recent years, advances in nanotechnology have resulted in the development of nanotools, capable of entering the single cell, resulting in new nanobiosensors useful for the detection of biomolecules inside living cells. In this paper, we provide an application of a nanotip coupled with molecular beacons (MBs) for the detection of DNA. The MBs were characterized by hybridization studies with a complementary target to prove their functionality both free in solution and immobilized onto a solid support. The solid support chosen as substrate for the immobilization of the MBs was a 30 nm tapered tip of an optical fiber, fabricated by chemical etching. With this set-up promising results were obtained and a limit of detection (LOD) of 0.57 nM was reached, opening up the possibility of using the proposed nanotip to detect mRNAs inside the cytoplasm of living cells.

## 1. Introduction

Usually, information on basic cellular signaling processes associated with diseases is not directly deducible from measurements on an average of a pool of cells. In fact, new information can only be obtained by monitoring the cellular signaling pathways directly inside intact cells [1]. Traditional approaches for studying cells and molecular biology outside living cells (e.g., by using biomolecules purified from cells), while being highly productive, often lose valuable information about cellular mechanisms that can only be observed in their natural environment. Hence, even if challenging, this progress in cellular physiology study requires new detection strategies at the nanoscale level applied to individual cells with large temporal and spatial resolution. In this framework, nanotechnology is becoming a key tool, opening new ways to explore and understand cellular and subcellular mechanisms [2]. In recent years, the advances in nanotechnology have led to the development of nanotools, capable of entering the single cell and detecting target biomolecules inside the living cells if coupled to the specific receptors. Examples of these nanotools are: fluorescent quantum dots, used to visualize dynamic processes in living cells [3,4,5]; carbon nanotubes or polymeric nanoparticles, used to penetrate the cell membranes as carrier of drugs or for imaging purpose [6,7,8]; gold nanorods, used for photothermal therapy [9]; and nanoneedles and nanotips which can penetrate the cell membranes with minimal cell damage [10,11].

Among these nanotools, nanoprobes based on fiber-optical nanotips were conceived as integrated nanodevices consisting of a recognition molecule coupled to the optical transducing element (the optical fiber) interfaced to a photo- or spectrometric detection system, aimed to perform diagnosis within single living cells [12,13,14,15].

Chemical nanosensors were first developed for monitoring calcium and nitric oxide, and other physiochemical parameters in single cells [16], showing excellent detection limits, as well as photostability, reversibility, and millisecond response. McCulloch *et al.* [17] prepared nanometric optical fiber sensors for intracellular pH measurements, while other groups [18] developed an optical fiber sensor for the measurement of dissolved oxygen based on sol-gel immobilization technology. Vo-Dinh and coworkers have developed nanobiosensors to detect biochemical targets inside living single cells [19,20,21,22], while Zhang [23] reported about an integrated device for optical stimulation and spatiotemporal electrical recording of neural activity in light-sensitized brain tissue.

In the field of DNA/RNA analysis in cell, MBs were proposed by Tyagi and Kramer [24,25,26,27,28,29]. MBs are single-stranded DNA molecules that possess a stem-loop structure commonly named hairpin structure. The loop portion of the molecule can form a double-stranded DNA in the presence of a complementary sequence. MB can be labeled with a fluorophore and a quencher at the two side-ends of the stem, constituted by a more or less long chain of complementary bases, which keeps these two moieties in close proximity to each other. Since the fluorophore is characterized by an emission band, which overlaps the absorption band of the quencher, this proximity causes the fluorescence of the fluorophore to be quenched by energy transfer. The fluorophore fluorescence is restored upon the opening of the stem due to the hybridization of the MB with the target sequence.

A great many fiber nanotips are characterized by a metal coating which allows them to illuminate exactly only the fiber nanotip. Actually, it is very difficult to quantify the exact fraction of light that will reach the nanotip since, below a certain inner diameter, even the lowest guided mode runs into cutoff, where the wave vector becomes imaginary and thus the mode field decays exponentially [30]. However, although the transmitted light fraction can be extremely small at the apex of the nanotip, when applying the nanoprobe for intracellular analysis, the dimensions of a single cell which are in the range 1 µm–10 µm, lead to the fact that the examined part of the nanoprobe will be not only the apex of 30 nm and consequently a larger portion of hundreds of nanometers will be exposed to the intracellular content. The absence of the metal coating on the fiber nanotip implies the excitation of the MB immobilized along the whole fiber nanotip. On the other hand, only the portion of the MB inside the cell which interacts with the intracellular target will emit fluorescence. A partial loss of localization associated with this approach can be accepted for some intracellular application where there is not a strong necessity of localization, if this is accompanied with an increase of the fluorescence signal associated to the excitation of a much larger number of molecules of MB.

**Figure 1:**
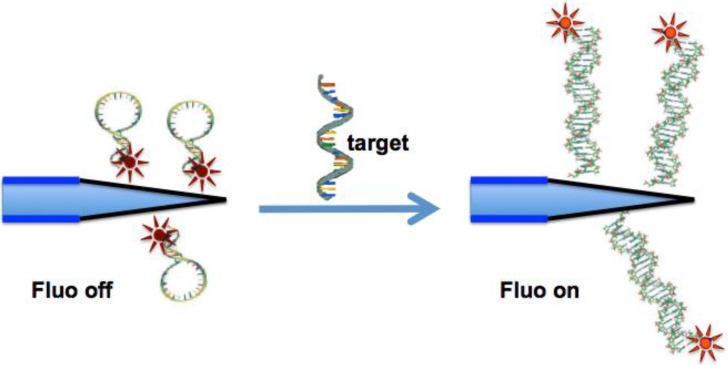
Scheme of the sensing mechanism.

In this paper we present preliminary results concerning the use of an optical fiber nanotip coupled to a MB for DNA detection (Figure 1). The solid support chosen as substrate for the immobilization of the MB was a tip of an optical fiber tapered at nanoscale size, intended to be used in the future for mRNA detection inside the cytoplasm of living cells. The nanotip was fabricated by chemical etching, starting from 500 micron—diameter of the multimode optical fiber—down to 30 nm at the tip. Then, the fiber tip was silanized, and the MB was attached via a covalent-binding procedure.

In particular, the attention was focused on the mRNA for survivin, a protein highly expressed in most types of cancer. In this case, the MB could act not only as detector of the over-expression of the mRNA for the survivin (diagnosis) but also as the blocking agent of the synthesis of the protein itself (therapy), as we demonstrated with Real-time PCR and western blotting experiments which showed a time-dependent reduction of survivin mRNA and protein after 100 nM-MB treatment, respectively, with the molecular beacon transfected into A375 melanoma cells [31,32].

After the characterization of the sole MB in solution carried out to verify the reliability and the effectiveness of the fluorophore/quencher pair immobilized at the extremities of the single-stranded DNA and at the same time of the good interaction of the MB with its target, a thorough opto-chemical characterization was performed in order to evaluate the possibility of getting a reliable, reproducible and robust system consisting of the MB immobilized onto the fiber nanotip.

## 2. Experimental Section

### 2.1. Chemicals

All the reagents for the preparation of phosphate buffered saline (PBS), 40 mM, pH 7.4; tris hydrochloride (tris-HCl), magnesium chloride (MgCl_2_) for the preparation of tris-buffer (10 mM tris-HCl, 10 mM MgCl_2_, pH 8); hydrofluoric acid (HF); hydrochloric acid (HCl); sulfuric acid (H_2_SO_4_); hydrogen peroxide (H_2_O_2_); methanol (MeOH) were purchased from Sigma-Aldrich (Milan, Italy). (3-Aminopropyl)triethoxysilane (APTES) was purchased by abcr GmbH (Karlsruhe, Germany). N-succinimidyl 3-(2-pyridyldithio) propionate (SPDP) was purchased from Pierce (Thermofisher Scientific, Milan, Italy). The molecular beacon for survivin, its complementary target sequence, the non-specific random sequence and the labeled linear probe were purchased from IBA (Gottingen, Germany).

### 2.2. Molecular Beacon and Target Sequences

The sequence for the survivin molecular beacon [31] was chosen among several published sequences [33,34,35,36] for its better performances in terms of folding properties and greater reactivity with the target sequence. A different fluorophore-quencher pair, ATTO647N (λ_abs_ 644 nm, λ_em_ 669 nm) and BlackBerry^®^ Quencher 650 (λ_max_ ~ 650 nm, useful absorbance between 550 and 750 nm), was chosen with respect to the one used for the published MBs in order to obtain a greater quantum yield (QY = 65 of the ATTO647N instead of 0.28 of the Cy5). The sequences of the different oligonucleotides (MB, specific target, random sequence and linear probe) are reported below:

MB     5'-(ATTO647N)CGACGGAGAAAGGGCTGCCACGXCG(BBQ)-3' X=C6-dT Thio
Target    5'-CCCCTGCCTGGCAGCCCTTTCTCAAGGACC-3'
Random sequence 5'-ATCGGTGCGCTTGTCG-3'
Linear probe  5'-(ATTO647N)GAGAAAGGGCTGCCA(Thiol)-3'

All the measurement carried out with both the MB in solution and the MB immobilized on the fiber tip were carried out at room temperature.

### 2.3. Optical Fiber Nanotip Preparation

The optical fiber nanotip was manufactured by a dynamic chemical etching method [37,38], by mechanically rotating and dipping a 3 M silica optical fiber, (core diameter 480 micron) in a chemical etching solution (aqueous hydrofluoric acid) covered with a protection layer.

Using different dynamic regimes of the mechanical movements during the chemical etching process, it was possible to vary the cone angle, the shape and the roughness of the nanoprobes. It was found that the tip profiles were determined by the nonlinear dynamic evolution of the meniscus of the etching solution near the fiber. Different regimes can be generated by changing the ratio between the angular velocities and the ratio between the radii of optical fiber and vial, ranging from laminar flow to the onset of chaotic flow. The type of flow of the viscous HF solution caused by the rotation of the vial and the optical fiber can be described in the framework of the Taylor-Couette flow (TCF) theory [39,40].

Moreover, by an accurate control of the extraction speed of the fiber from the HF solution, the length and the angle of the nanotip were precisely controlled, and the capillarity effects at the nanoscale were minimized by limiting strong friction on the optical fiber nanotip in the final part of the etching process.

**Figure 2:**
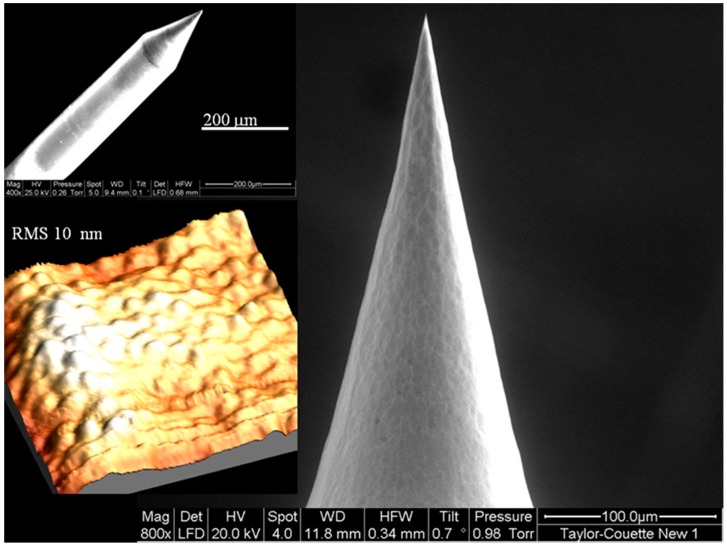
Example of a nanotip obtained using the fabrication method described in Section 2.3. In the left top a general view showing a taper length of about 200 µm and a large cone angle (about 40°), while in the left bottom an AFM image (10 µm × 10 µm) is reported, with an estimated roughness of about 10 nm. On the right panel is reported a zoom on the distal end, showing an apex angle of about 24°.

SEM pictures and AFM measurements have been performed on different nanotips showing our ability to reproduce typical tip features, such as short taper length (~200 μm), large cone angle (from 15° to 40°), small probe tip dimension (less than 30 nm), and roughness below 10 nm. The geometrical characterization demonstrated that, with this method, a high yield of reproducible nanotips can be obtained, overcoming some drawbacks of conventional etching techniques. The nanometric roughness is the key point to keep the scattering, which can perturb the measurements, at the lowest levels. Figure 2 shows an example of nanotip obtained using the fabrication method described above.

In particular, in the left top part a general view of the short taper length and the large cone angle (about 40° in this example) of the nanotip is shown, while in the left bottom an AFM image (10 µm × 10 µm) is reported, with an estimated roughness of the tip of about 10 nm. On the right panel is reported a zoom on the distal end, showing the apex angle (about 24°).

The glass nanotip was dipped in piranha solution (H_2_SO_4_:H_2_O_2_ 7:3) for 30 min to carry out a strong cleaning and to activate the silanol groups present on the surface. Then the tapered fiber nanotip was functionalized by a 2-hour long silanization procedure using 5% APTES in MeOH/H_2_O (1:1) in order to achieve the amino groups onto the surface of the nanotip. After several rinsing cycles in MeOH and deionized water, the nanotip was cured in oven at 100 °C for 1 h.

SPDP was used as crosslinker between the -NH_2_ groups available onto the nanotip and the -SH groups of the linear probe or of the MB. After the treatment with a solution of SPDP 4 mM in PBS at pH 7.4 for 30 min, the nanotip was rinsed in PBS and then in tris-buffer. As already reported by Tombelli *et al* [41], the cross-linker SPDP could be used to form a cleavable disulphide bond to detach the MB from the delivery vehicle inside the cytoplasm. This could be an advantage if the aim is to release the MB inside the cells, and, consequently the nanotip is used simply as the delivery tool of the MB as therapeutic molecule inside the cell. On the other hand, if the probe is used as imaging tool, other cross-linkers, such as succinimidyl-4-(N-maleimidomethyl)cyclohexane-1-carboxylate (SMCC) should be considered in order to avoid any loss of signal caused by the MB detachment from the fiber nanotip.

Before its immobilization, the MB was heated for 5 min in water bath at about 70–80 °C (slightly higher than the melting temperature) and then left to slowly equilibrate down to room temperature in order to optimize the hairpin formation. The immobilization of 1 µM MB (in tris-buffer), or of 1 µM linear probe, onto the nanotip was performed over night at 4 °C.

### 2.4. The Optical Measurements

Scanning Electron Microscope (SEM, Quanta-200 FEI, Hillsboro, OR, USA) was used to characterize the geometrical features of the nanotips, whereas the surface roughness was checked by Atomic Force Microscopy (AFM, prototype developed by Pini *et al.* [42]).

**Figure 3:**
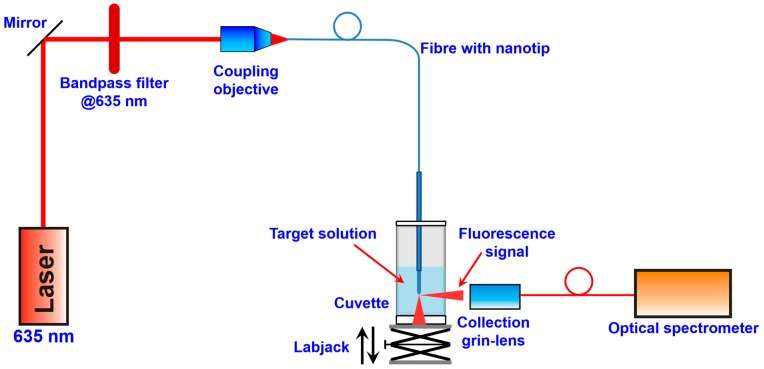
Optical experimental set-up.

An in-house optical set-up, illustrated in Figure 3, was used to characterize the fluorescent nanoprobes. Fluorescence measurements on fiber nanotips were carried out in a cuvette using, for the MB excitation, a LDH-P-C-635B laser diode (PicoQuant, Berlin, Germany) emitting at 635 nm filtered with a bandpass interference filter (FL635-10, ThorLabs, Newton, NJ, USA). The emitted fluorescence was collected by means of a GRIN lens coupled with a multimode optical fiber (diameter 200 μm), aligned with the fiber nanotip and then guided to an optical high-pass filter Thorlabs FEL0650, in order to filter the excitation light scattered out from the fiber tip, and finally acquired by a Shamrock 303i spectrograph (Andor, Belfast, United Kingdom). The cuvette was fixed inside a suitable holder onto a manual labjack (model 271 Labjack, Newport, Irvine, CA, USA) and the fiber was immersed into the cuvette from the top. In this way, it was possible, after the optical alignment, to move down the cuvette, to empty and fill it or to exchange the cuvette, and to move up again it to the exact same position, without changing the alignment of the nanotip and GRIN lens.

The experimental set-up for fluorescence measurement of the molecular beacon in solution was very similar to the one described in Figure 3, with the difference that the fluorescent solution into the cuvette was excited directly with the collimated laser beam, without the objective, and collected with the GRIN lens in normal direction with respect to the excitation laser beam direction. The measurements in solution were performed without the optical high-pass filter Thorlabs FEL0650, because of the better signal (fluorescence) to noise (scattered excitation light) ratio of this measurement configuration.

## 3. Results and Discussion

### 3.1. MB Characterization in Solution

The functionality of the MB was firstly investigated in buffer solution by hybridization with the specific target at different incubation times. Different buffers and different culture media were already tested and reported, such as Dulbecco’s modified Eagle’s Medium (DMEM) [43,44]. Even if tris-buffer is not mimicking the environmental condition within the cell, it is the one which provides the best performances of the molecular beacon in solution.

**Figure 4:**
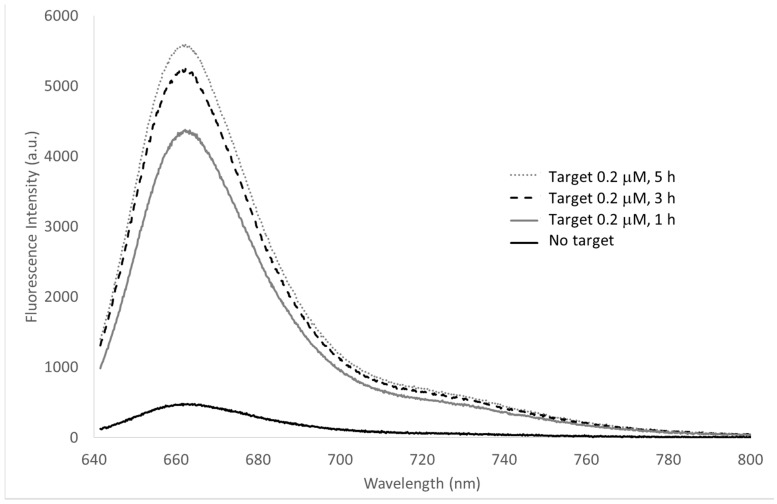
Fluorescence spectra of the molecular beacon (0.1 µM) after incubation for 1, 3 and 5 h with the specific target 0.2 µM. λ_ex_ 635 nm, exposure time 1 s.

The MB was characterized by recording the fluorescence (λ_ex_ 635 nm) of the MB 100 nM in Tris buffer alone and after 1, 3 and 5 h of incubation with the specific target 0.2 µM (Figure 4). A final 10-times increase of the MB fluorescence in presence of the target was recorded with the signal increasing with time from 1 to 5 h. The characterization on such a long time, much longer than the interaction time between the MB and the target, was carried out in order to verify the stability of the molecular beacon and, consequently, of its emitted fluorescence also after that the interaction took place.

### 3.2. MB and Linear Probe Characterization onto the Nanotip

**Figure 5:**
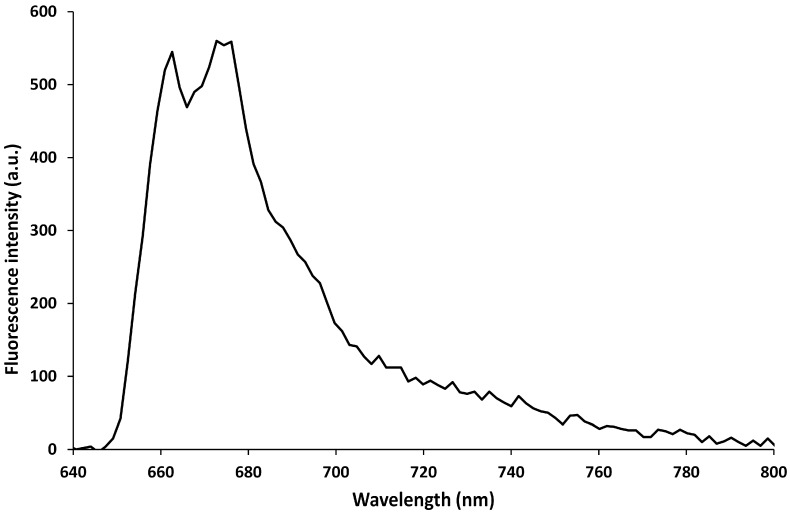
Fluorescence spectra of the linear probe immobilized onto the nanotip. λ_ex_ 635 nm, exposure time 1 s.

Before working with the molecular beacon, the fluorescent linear probe was immobilized onto the nanotip in order to evaluate the actual possibility of sensing the presence of a labeled oligonucleotide by direct excitation of the oligonucleotide fluorophore by means of the same fiber nanotip. After the probe immobilization, the fluorescence of the ATTO647N fluorophore labeling the probe, was measured (λ_ex_ 635 nm). Figure 5 shows the fluorescence spectrum of the linear probe immobilized onto the nanotip. The fluorescence peak shown in the Figure is split into two peaks, at 660 nm and 680 nm due to the not uniform transmission spectrum of the Thorlabs FEL0650 high-pass filter.

The fluorescence signal of the probe was monitored for 30 min in order to evaluate the fluorescence stability: the average fluorescence intensity in 30 min was of 11,300 (a.u.) with a standard deviation of 436 (a.u.) leading to a signal variability of 3.8%.

**Figure 6:**
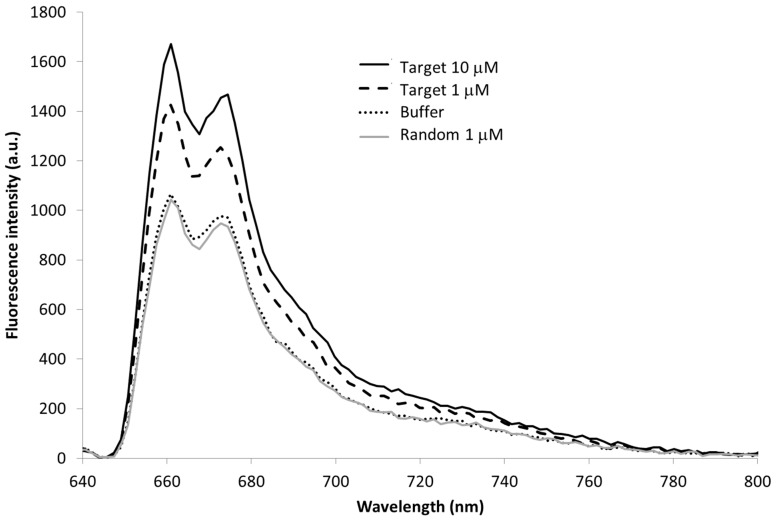
MB immobilized onto the nanotip, characterization in presence of the target (1 µM and 10 µM) and of the random sequence (1 µM). λ_ex_ 635 nm, exposure time 1 s.

In Figure 6 the fluorescence spectrum of the multimode fiber functionalized with the molecolar beacon is shown. In particular, the nanotip was immersed in pure buffer (Tris), in a solution of the random sequence and in two solutions of the specific target 1 and 10 µM and the fluorescence spectra were recorded after 30 min.

**Figure 7:**
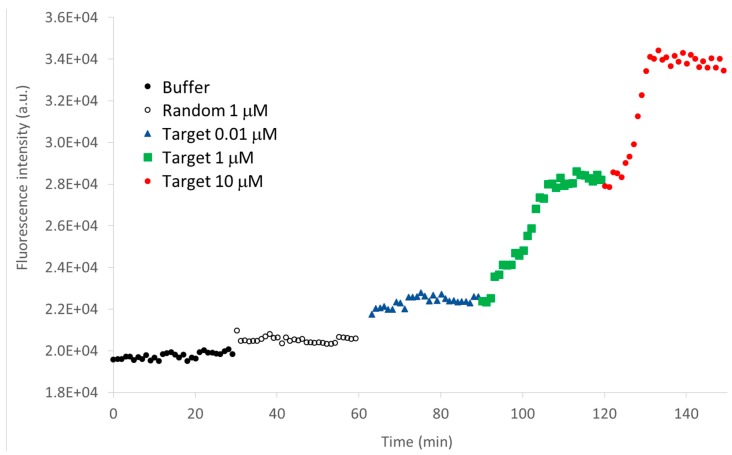
Fluorescence signal (*vs.* time) during the interaction of the MB immobilized onto the nanotip with the random sequence, and the target at different concentrations (0.01 µM, 1 µM and 10 µM).

The performance of the fiber nanotip to act as sensor was evaluated by monitoring the fluorescence signal as a function of time, when exposed to different concentrations of the target. Its specificity when interacting with the random sequence was also investigated (Figure 7). In particular, after an initial stabilization of the MB fluorescence in buffer, the coated nanotip was first immersed in a solution 1 µM of the random sequence and the fluorescence was recorded for 30 min. Thereafter, the same nanotip was consecutively incubated for the same time in target solutions at different concentrations. As can be seen from the figure, only a 3.5% fluorescence increase was observed in presence of the random sequence, whereas the increase with only 0.01 µM of the target was of about 13%. Each point of the graph is evaluated as the sum of the optical intensity detected by the optical spectrograph between 650 and 720 nm.

The curve in Figure 7 well testifies for the feasibility of the MB-coated nanotip to act as a sensor, providing also information on the kinetics of the interaction between the MB and the target. The nanotip was exposed to the different solutions (random and target solutions) for 30 min in order to perform an opto-chemical characterization, even if the timing needed was showed to be minor than 10 min.

**Figure 8:**
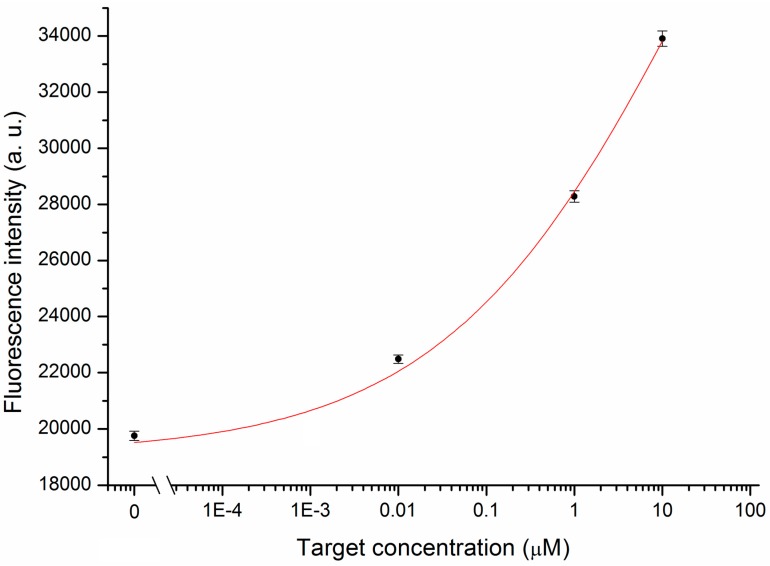
Calibration curve obtained from the interaction of the MB-coated nanotip with increasing concentrations of target.

Figure 8 shows the calibration curve obtained by averaging the experimental data at the plateau of the binding curve and calculating the related standard deviation for each target concentration. A Limit of Detection (LOD) of 0.57 nM for the proposed sensor was calculated from the calibration curve considering three times the greater difference between the experimental data and the fitting curve (maximum of residuals).

The limit of detection achieved opens up the possibility of using the proposed nanotip to detect mRNAs inside the cytoplasm of living cells. In fact, the concentration of specific mRNAs has been estimated to be in the nM range [10], although it is quite difficult to estimate the mRNA concentration inside the cytoplasm since it is not homogeneously distributed, but it has preferential locations. In the case of survivin mRNA, which is the molecule of interest, preferential localization is at the perinuclear position [34].

**Figure 9:**
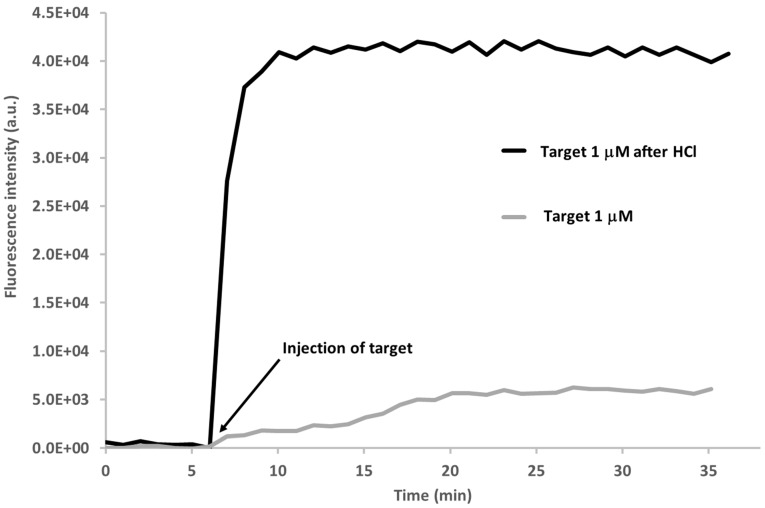
Kinetics of the fluorescence signal of the MB onto the nanotip after incubation with the target 1 µM without any previous treatment and after the treatment with HCl 2 mM.

By investigating the possibility of regenerating the nanotip for multiple use by the use of HCl, we discovered that it is possible to increase the performance of the sensor. Figure 9 shows the comparison between the response of the fiber nanotip to the 30 min exposure to 1 µM target solution (grey tracing) shown in Figure 7 (green tracing) and the response to the same target concentration after having exposed the fiber nanotip to HCl 2 mM for 1 min (black tracing). In this graph, the baselines of the two curves were both moved to the value of zero for a better comparison. After the HCl treatment, the nanotip was thoroughly washed in tris-buffer and left to equilibrate in this buffer until a stable fluorescence signal was reached, before exposing it to the target solution.

An 8-fold increase in the fluorescence signal due to the interaction of the MB with the target can be observed. This behaviour can be ascribed to the unfolding effect of HCl which is able to open the hairpin structure of the MB and the subsequent refolding of the beacon hairpin structure in a more efficient manner with respect to the untreated beacon which could be also folded around the nanotip and consequently hindered towards the hybridisation with the target. This fact can provide the explanation of the faster kinetics which is observed after the HCl treatment, with the plateau reached after only 4 min, instead of 15 min. The use of HCl 1–2 mM, among others regenerating solutions, is quite common in DNA biosensor regeneration [45,46,47]. The demonstration that the MB is not degraded by this treatment is given by the high and fast fluorescent increase from PBS (target zero) to the target 1 μM, as shown in Figure 8. The HCl treatment is therefore essential in order to achieve a correct and ready-to-interact MB layer on the fiber nanotip immediately after its immobilization which can occur also with secondary structures hampered towards the proper interaction with the target. The same treatment could also be used in the regeneration of the sensor after the interaction with the target in order to remove the target from the MB and make it fold again in its closed structure ready for a new hybridisation cycle.

## 4. Conclusions

The present paper describes the manufacture and optical characterization of an optical fiber nanotip coated with molecular beacons potentially capable to perform intracellular measurements. The optical characterization showed the good performance of the obtained nanotip in terms of selectivity and sensitivity, and a limit of detection of 0.57 nM was achieved. The treatment with HCl, besides allowing a regeneration of the nanotip, is able to provide a better performance of the molecular beacon with a further decrease of the limit of detection.

## References

[1] Vo-Dinh T. (2008). Nanosensing at the single cell level. Spectrochim. Acta Part B At. Spectrosc..

[2] Zheng X.T., Hu W., Wang H., Yang H., Zhou W., Li C.M. (2011). Bifunctional electro-optical nanoprobe to real-time detect local biochemical processes in single cells. Biosens. Bioelectron..

[3] Dahan M., Lévi S., Luccardini C., Rostaing P., Riveau B., Triller A. (2003). Diffusion dynamics of glycine receptors revealed by single-quantum dot tracking. Science.

[4] Pathak S., Cao E., Davidson M.C., Jin S., Silva G.A. (2006). Quantum Dot Applications to Neuroscience: New Tools for Probing Neurons and Glia. J. Neurosci..

[5] Ma Q., Lin Z., Yang N., Li Y., Su X.-G. (2014). A novel carboxymethyl chitosan–quantum dot-based intracellular probe for Zn^2+^ ion sensing in prostate cancer cells. Acta Biomater..

[6] Duchi S., Sotgiu G., Lucarelli E., Ballestri M., Dozza B., Santi S., Guerrini A., Dambruoso P., Giannini S., Donati D. (2013). Mesenchymal stem cells as delivery vehicle of porphyrin loaded nanoparticles: Effective photoinduced *in vitro* killing of osteosarcoma. J. Control Release.

[7] Giannetti A., Tombelli S., Baldini F. (2013). Oligonucleotide optical switches for intracellular sensing. Anal. Bioanal. Chem..

[8] Sokolova V., Epple M. (2008). Inorganic Nanoparticles as Carriers of Nucleic Acids into Cells. Angew. Chem. Int. Ed..

[9] Ratto F., Matteini P., Centi S., Rossi F., Pini R. (2010). Gold nanorods as new nanochromophores for photothermal therapies. J. Biophoton..

[10] Kihara T., Yoshida N., Kitagawa T., Nakamura C., Nakamura N., Miyakea J. (2010). Development of a novel method to detect intrinsic mRNA in a living cell by using a molecular beacon-immobilized nanoneedle. Biosens. Bioelectron..

[11] Yum K., Wang N., Yu M.-F. (2010). Nanoneedle: A multifunctional tool for biological studies in living cells. Nanoscale.

[12] Dufrêne Y., Garcia-Parajo M.F. (2012). Recent progress in cell surface nanoscopy: Light and force in the near-field. Nano Today.

[13] Zheng X.T., Li C.M. (2010). Single living cell detection of telomerase over-expression for cancer detection by an optical fibre nanobiosensor. Biosen. Bioelectron..

[14] Zheng X.T., Yang H.B., Li C.M. (2010). Optical Detection of Single Cell Lactate Release for Cancer Metabolic Analysis. Anal. Chem..

[15] Vitol E.A., Orynbayeva Z., Friedman G., Gogotsi Y. (2012). Nanoprobes for intracellular and single cell surface-enhanced Raman spectroscopy (SERS). J. Raman Spectrosc..

[16] Brasuel M., Kopelman R., Kasman I., Miller T.J., Philbert M.A. (2002). Ion Concentrations in Live Cells from Highly Selective Ion Correlations Fluorescent Nano-Sensors for Sodium. IEEE Proc..

[17] McCulloch S., Uttamchandani D. (1997). Development of a fibre optic micro-optrode for intracellular pH measurements. IEE Proc. Optoelectron..

[18] Xu H., Aylott J.W., Kopelman R., Miller T.J., Philbert M.A. (2001). A Real-Time Ratiometric Method for the Determination of Molecular Oxygen Inside Living Cells Using Sol−Gel-Based Spherical Optical Nanosensors with Applications to Rat C6 Glioma. Anal. Chem..

[19] Vo-Dinh T., Alarie J.P., Cullum B.M., Griffin G.D. (2000). Antibody-based nanoprobe for measurement of a fluorescent analyte in a single cell. Nat. Biotechnol..

[20] Vo-Dinh T., Kasili P. (2005). Fibre-optic nanosensors for single-cell monitoring. Anal. Bioanal. Chem..

[21] Vo-Dinh T., Kasili P., Wabuyele M. (2006). Nanoprobes and nanobiosensors for monitoring and imaging individual living cells. Nanomed. Nanotechnol. Biol. Med..

[22] Zhang Y., Dhawan A., Vo-Dinh T. (2011). Design and Fabrication of Fibre-Optic Nanoprobes for Optical Sensing. Nanoscale Res. Lett..

[23] Zhang J., Laiwalla F., Kim J.A., Urabe H., Van Wagenen R., Song Y.-K., Connors B.W., Zhang F., Deisseroth K., Nurmikko A.V. (2009). Integrated device for optical stimulation and spatiotemporal electrical recording of neural activity in light-sensitized brain tissue. J. Neural Eng..

[24] Tyagi S., Kramer F.R. (1996). Molecular beacons: Probes that fluoresce upon hybridization. Nat. Biotechnol..

[25] Liu X., Farmerie W., Schuster S., Tan W. (2000). Molecular Beacons for DNA Biosensors with Micrometer to Submicrometer Dimensions. Anal. Biochem..

[26] Monroy-Contreras R., Vaca L. (2011). Molecular Beacons: Powerful Tools for Imaging RNA in Living Cells. J. Nucleic Acids.

[27] Santangelo P.J. (2010). Molecular beacons and related probes for intracellular RNA imaging. Wiley Interdiscip. Rev. Nanomed. Nanobiotechnol..

[28] Wang Q., Chen L., Long Y., Tian H., Wu J. (2013). Molecular Beacons of Xeno-Nucleic Acid for Detecting Nucleic Acid. Theranostics.

[29] Boutorine A.S., Novopashina D.S., Krasheninina O.A., Nozeret K., Venyaminova A.H. (2013). Fluorescent Probes for Nucleic Acid Visualization in Fixed and Live Cells. Molecules.

[30] Hecht B., Sick B., Wild U.P., Deckert V., Zenobi R. (2000). Scanning near-field optical microscopy with aperture probes: Fundamentals and applications. J. Chem. Phys..

[31] Carpi C., Fogli S., Giannetti A., Adinolfi B., Tombelli S., Da Pozzo E., Vanni A., Martinotti E., Martini C., Breschi M.C. (2014). Theranostic properties of a survivin-directed molecular beacon in human melanoma cells. PLoS ONE.

[32] Adinolfi B., Carpi S., Giannetti A., Nieri P., Pellegrino M., Sotgiu G., Tombelli S., Trono C., Varchi G., Baldini F. (2014). Complex nanostructures based on oligonucleotide optical switches and nanoparticles for intracellular mRNA sensing and silencing. Proc. Eng..

[33] Nitin N., Santangelo P.J., Kim G., Nie S., Bao G. (2004). Peptide-linked molecular beacons for efficient delivery and rapid mRNA detection in living cells. Nucleic Acids Res..

[34] Santangelo P.J., Nix B., Tsourkas A., Bao G. (2004). Dual FRET molecular beacons for mRNA detection in living cells. Nucleic Acids Res..

[35] Qiao G., Gao Y., Li N., Yu Z., Zhuo L., Tang B. (2011). Simultaneous Detection of Intracellular Tumor mRNA with Bi-Color Imaging Based on a Gold Nanoparticle/Molecular Beacon. Chem. Eur. J..

[36] Xue Y., An R., Zhang D., Zhao J., Wang X., Yang L., He D. (2011). Detection of survivin expression in cervical cancer cells using molecular beacon imaging: New strategy for the diagnosis of cervical cancer. Eur. J. Obstet. Gynecol. Reprod. Biol..

[37] Barucci A., Cosi F., Pelli S., Soria S., Nunzi Conti G., Giannetti A., Righini G. (2014). Method of Fabricating Structures, Starting from Material Rods. Patent Pending.

[38] Barucci A., Cosi F., Giannetti A., Pelli S., Griffini D., Insinna M., Salvadori S., Tiribilli B., Righini G.C. (2015). Optical fibre nanotips fabricated by a dynamic chemical etching for sensing applications. J. Appl. Phys..

[39] Taylor G.I. (1923). Stability of a Viscous Liquid Contained between Two Rotating Cylinders. Philosoph. Trans. R. Soc. London Ser. A Contain. Pap. Math. Phys. Charact..

[40] Andereck C.D., Liu S.S., Swinney H.L. (1986). Flow regimes in a circular Couette system with independently rotating cylinders. J. Fluid Mech..

[41] Tombelli S., Ballestri M., Giambastiani G., Giannetti A., Guerrini A., Sotgiu G., Trono C., Tuci G., Varchi G., Baldini F. (2013). Oligonucleotide switches and nanomaterials for intracellular mRNA sensing. Proc. SPIE.

[42] Pini V., Tiribilli B., Gambi C.M.C., Vassalli M. (2010). Dynamical characterization of vibrating AFM cantilevers forced by photothermal excitation. Phys. Rev. B..

[43] Giannetti A., Baldini F., Ballestri M., Ghini G., Giambastiani G., Guerrini A., Sotgiu G., Tombelli S., Trono C., Tuci G. (2014). Intracellular nanosensing and nanodelivery by PMMA nanoparticles. Lect. Notes Electri. Eng..

[44] Giannetti A., Tombelli S., Trono C., Ballestri M., Giambastiani G., Guerrini A., Sotgiu G., Tuci G., Varchi G., Baldini F. (2013). Intracellular delivery of molecular beacons by PMMA nanoparticles and carbon nanotubes for mRNA sensing. Proc. SPIE..

[45] Lechuga L.M. New frontiers in optical biosensing. Proceedings of the 13th European Conference on Integrated Optics (ECIO 2007).

[46] Mannelli I., Minunni M., Tombelli S., Wang R., Spiriti M.M., Mascini M. (2005). Direct immobilization of DNA probes for the development of affinity biosensors. Bioelectrochemistry.

[47] Dos Santos Riccardi C., Yamanaka H., Josowicz M., Kowalik J., Mizaikoff B., Kranz C. (2006). Label-free DNA detection based on modified conducting polypyrrole films at microelectrodes. Anal. Chem..

